# Cascades in capacity constrained agents

**DOI:** 10.1371/journal.pone.0280326

**Published:** 2023-01-20

**Authors:** Jacob Derechin

**Affiliations:** Department of Sociology, Yale University, New Haven, Connecticut, United States of America; Universita degli Studi di Siena, ITALY

## Abstract

Many sorts of contagious phenomenon, such as music, do not exist in isolation but as part of a competitive marketplace. In these settings there are often superstars with out-sized popularity along with a large number of flops with little popularity. It could be the case that superstars are more popular because they are higher quality but I suggest that capacity constraints may be a structural factor that influences these disparities. In this agent-based model, there are multiple potentially cascading states that the agent can potentially occupy. The agents have a certain capacity of states that they can occupy at once. For example, suppose someone has a workout playlist that lasts 1 hour. As they discover new music to add to the playlist, they have to remove songs currently in the playlist to keep the playlist 1 hour. Thus, in this setting, the states indirectly trade off with each other by virtue of the capacity constraint. The key question is whether the indirect trade offs imposed by the capacity constraint are enough to induce disparities in popularity, even when the states are otherwise identical. I find that increasing the number of states in excess of capacity increases the disparities between popular and unpopular states. This suggests that capacity constraints may be a structural factor in explaining market concentration and superstar phenomenon.

## Introduction

Diffusion processes are an important social phenomenon that has been studied across multiple domains such as consumer goods [1], adoption of hybrid corn [2], the spread of disease [3], information on social media [4], revolutions [5], as well as many theoretical explorations [6–11].

Cascades often do not occur in isolation, but exist in an environment with multiple potential cascades that could occur simultaneously. One way to model multiple simultaneous cascades or contagions is through direct interaction between the contagions. These models are typically used in an epidemiological setting where there is some sort of infection that is spreading and a social behavior like vaccination or distancing also spreads through the population, which modulates the spread of the infection but is also driven by the infection [12–15]. The interaction between multiple contagions can become computationally challenging but in well mixed populations it has been shown that these models are equivalent to complex contagion models [16].

Simultaneous cascade models can help clarify why some things before popular while others do not. The first class of explanations is around quality: things are popular because they are better. The implication here is that popularity is proportional to quality, so if one musician sells 100 times more tickets than another one would expect their music to be 100 times better. The second class of explanations is based on market structure: things are popular because they are somehow advantaged. The implication here is that one would be able to find components of the market structure that could generate disparities in popularity even among identical products. On way to think about identical products is: products which can be differentiated via a label but are identical in use (generic drugs vs name brand drugs in a world where they are also the same price).

When considering identical valued products, it is natural to think of them as substitutes for each other. For example no matter how many washing machine brands are on the market, I as a consumer only need one washing machine and would be unlikely to buy two washing machines of different brands. Thus once my capacity for washing machines is met, I have no need to buy any more washing machines. While products that someone only needs one of can easily demonstrate the capacity constraint, the capacity for other products could be greater than one. For example one could regularly listen to a playlist of songs that lasts 1 hour, changing out the different songs over time as their tastes change instead of adding new songs to the end. It it also reasonable that different people could have different capacities for the same product, for example a family of four probably wants more spoons than an individual who lives alone does or different people could be perscribed different doses of the same drug. Given the similarities between this process and the types of congestion White observed in human communication networks, it is reasonable to expect these kinds of dynamics to influence a wide range of products [17].

Models of Oligopolistic competition can be a good way to undestand the market dymanics of identical products. The classic comparison would be between the Cournot Model (simultaneous output choice) and the Stackelberg model (sequential output setting), suggesting that the structural advantage of being able to set prices first drives a disparity between the equilibrium profits of the firms [18, 19]. In this way, capacity constraints could provide a sort of incumbency advantage to products which are adopted first and could serve to add Stackelberg like dynamics to diffusion models.

Given that the music market regularly experiences multiple overlapping cascades, the stylized characteristics of the market can be useful for understanding how multicascade processes empirically function. Notably the music market is somewhere one would expect to find elements of both quality driving popularity and structural popularity. On a structural level, the music industry historically has experienced high levels of firm concentration and this concentration is associated with a lower level musical diversity [20, 21]. At the level of artists the music industry also shows signs of concentration where a small number of popular artists have out sized influence at any given time [22–24]. Quality in music has proven difficult to empirically measure, as experimental evidence suggests that social influence and more typical cascade dynamics are at play [25, 26], but more recent analysis suggests that these effects may only be temporary perturbations from songs’ fundamental value [27]. In their experiment [26] presented their participants with 48 different songs to potentially listen to and download for free and found that they listened to only 7 songs on average only downloaded 1 on average. This suggests that the their participants may be capacity constrained in both their interest in listening to songs and downloading them.

## Materials and methods

### Model

This model is a variation of the Threshold Cascade model as described in [28]. There are N agents in the population who each have a cascade capacity of C. This means that if that agent would adopt a cascading state that would bring the number of states adopted greater than C, one of the currently adopted states is randomly dropped. There are S total states that can cascade and in order for the capacity constraint to be binding *S* > *C*. Agents can adopt a new state through either Threshold Cascading or Random adoption. Threshold cascading follows the process described in [7], each agent has a threshold *T*_*i*_ and adopts the state if the number of agents who have already adopted the state is greater than or equal to *T*_*i*_. Each state is evaluated independently of each other state, so an agent would not consider the number of agents who adopted state 1 when evaluating state 2. The threshold *T*_*i*_ varies across agents, but it does not vary between states. As in [7], the thresholds drawn from a normal distribution with mean *μ* and variance *σ*. Since the number of states adopted is discrete, the values of output by the normal distribution were rounded. For Random adoption, each agent is challenged each time step to adopt a random state. If the agent has already adopted the state, nothing changes. If the agent has not already adopted the state it adopts the state with probability p as defined by $p=\frac{1}{1+e^{mT_{i}}}$. A similar formulation for random adoption was used by [28]. At each time step each agent first checks for threshold adoption, then random adoption and finally checks for capacity. Since states are randomly dropped at the end of the round, it is possible for a state to be adopted and dropped within the same round.

When the model is initialized all agents are assigned their threshold but begin having adopted no states. This means that in order for a state to spread via threshold contagion it must first be randomly adopted. All agents have access to the set of states every other agent has adopted at the end of the round (after dropping for capacity) and uses this to determine threshold adoption in the next round. The variation in adoption thresholds, *T*_*i*_, among the agents represents the key structure in this population. Therefore one could interpret the threshold contagion model as a form of complex contagion model, but on a complete graph [29]. Instead of focusing on spread between individuals this model is better suited for describing population level phenomenon. While it is true than individual and network level dynamics can matter, the presence of small global signals can overpower local diffusion [30].

There have been some attempts to quantify thresholds at the individual and network level [31, 32], but attempts to merge threshold models with more classical diffusion models have also been successful [33, 34]. This suggests that even while thresholds may be difficult to empirically measure the results of these models can be compared to other classes of diffusion models.

### Parameter space

This model has 7 parameters. N represents the total number of agents in the population. T represents the total number of time steps. m represents the slope parameter in the random adoption function. C represents the capacity of each agent. S represents the total number of states. *μ* represents the mean of the threshold distribution and *σ* represents the variance of the threshold distribution. Table 1 shows the ranges of each of the parameters contained in this experiment.

**Table 1:** Parameter space.

	Range
N	100
T	100
m	0.2
C	3,4, (5,10,15,20)
S	5,10,15,20
*μ*	0–100
*σ*	0–100

Since this simulation is designed to test the effect of imposing the capacity constraint it was designed similarly to a randomized control trial with a control arm and two treatment arms. The first arm represents the control, with no capacity constraints. In this arm capacity was set to the number of states, so when states was 5 capacity was 5 and so on. The next arm is a treatment arm, where capacity was set to 3. In this arm there are excess capacities of 2,7,12, and 17. The final arm is a treatment arm where capacity was set to 4. In this arm there are excess capacities of 1,6,11, and 16.

There are 122412 different combinations in this parameter space and each unique parameter combination was replicated 100 times so there were 12241200 total runs in this experiment. This experiment was constructed using the python package AgentPy. [35]

## Results

### Outcomes of interest

The core outcome of interest is the number of agents who have adopted a given state. Going forward I will refer to one agent adopting a state at a given time as a count. Since counts vary both over time within a simulation run and across states, I need to aggregate in two dimensions. To aggregate across time, I look at the average number of counts over time (average counts), variance in counts over time (variance in counts), the maximum count reached (max counts), the time to reach the maximum (time to max), the sum of counts over time (final counts), the minimum count after the maximum count was reached (min after max), the time between the maximum and the minimum after maximum (decay time), as well as the Shannon entropy [36] in counts (entropy counts). Once these aggregations across time are calculated for each state, I aggregate across state using the mean, median, variance, mean absolute deviation, maximum and range (maximum—minimum).

It is also important to measure concentration/ tailedness in the distributions across states. To do this, I also the kurtosis, the Herfindahl-Hirschman Index [37, 38] across final counts and number of states where final count is zero. Since these are measuring concentration, final counts (sum over time) is the natural way to aggregate over time. Table 2 shows the summary statistics for these outcomes.

**Table 2:** Summary statistics.

	Mean	Min	Max	Standard Deviation
Final Counts	628.0637	0	9900	1811.4700
Max Counts	12.1457	0	100	20.6931
Time to Max	25.3517	0	99	30.0161
Average Counts	9.7456	0	99	17.6240
Variance in Counts	41.9046	0	2441.5454	172.7186
Min after Max	10.4242	0	100	19.6381
Decay Time	5.1842	0	98	12.4100
Entropy in Counts	4.5682	0	19	4.6561
Herfindahl Index	0.1445	0	1	0.1429
Kurtosis in Counts	0.7139	-3.3333	20	2.9554
Number of Zero Counts	1.7652	0	20	4.6101

**Fig 1:**
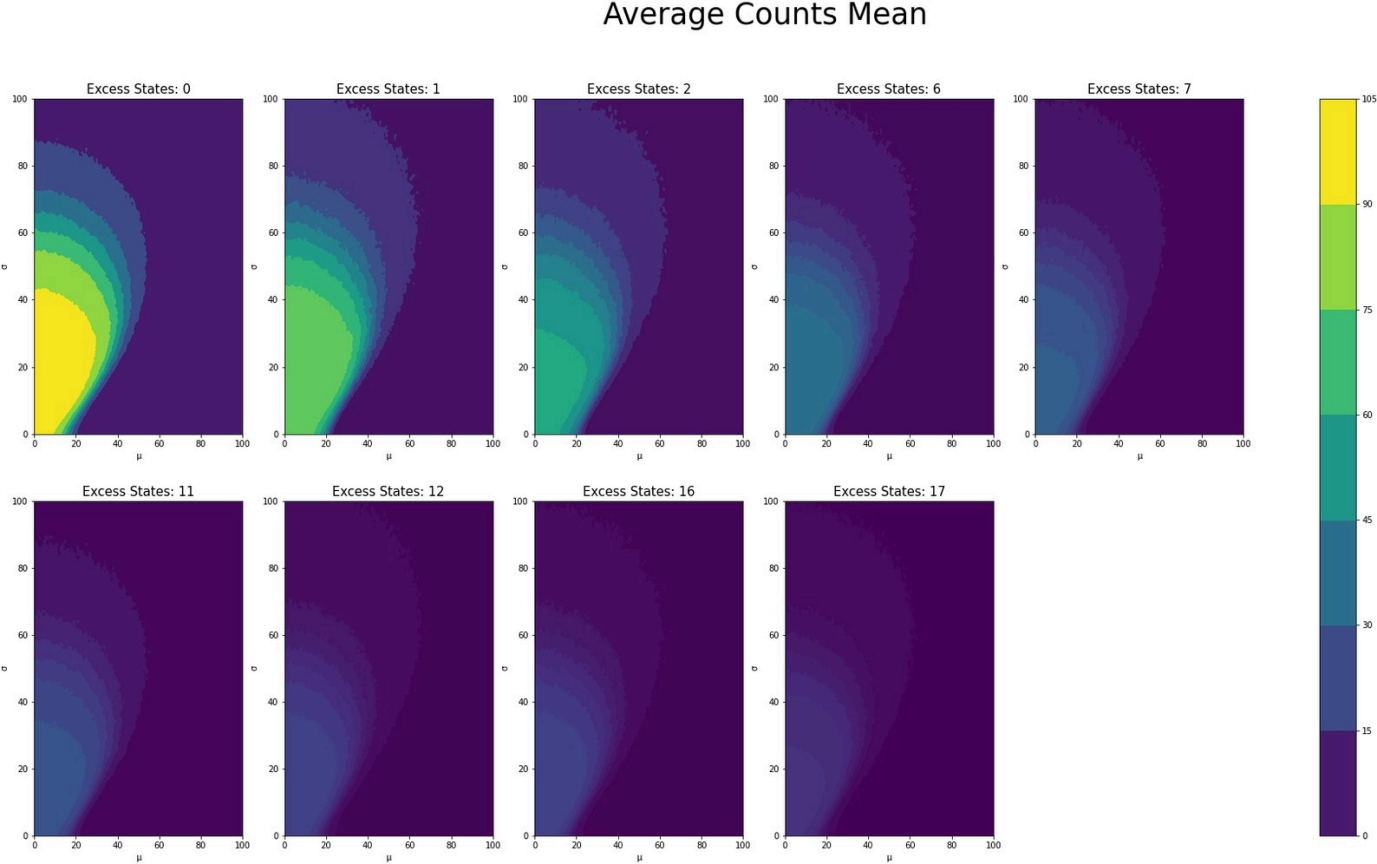


**Fig 2:**
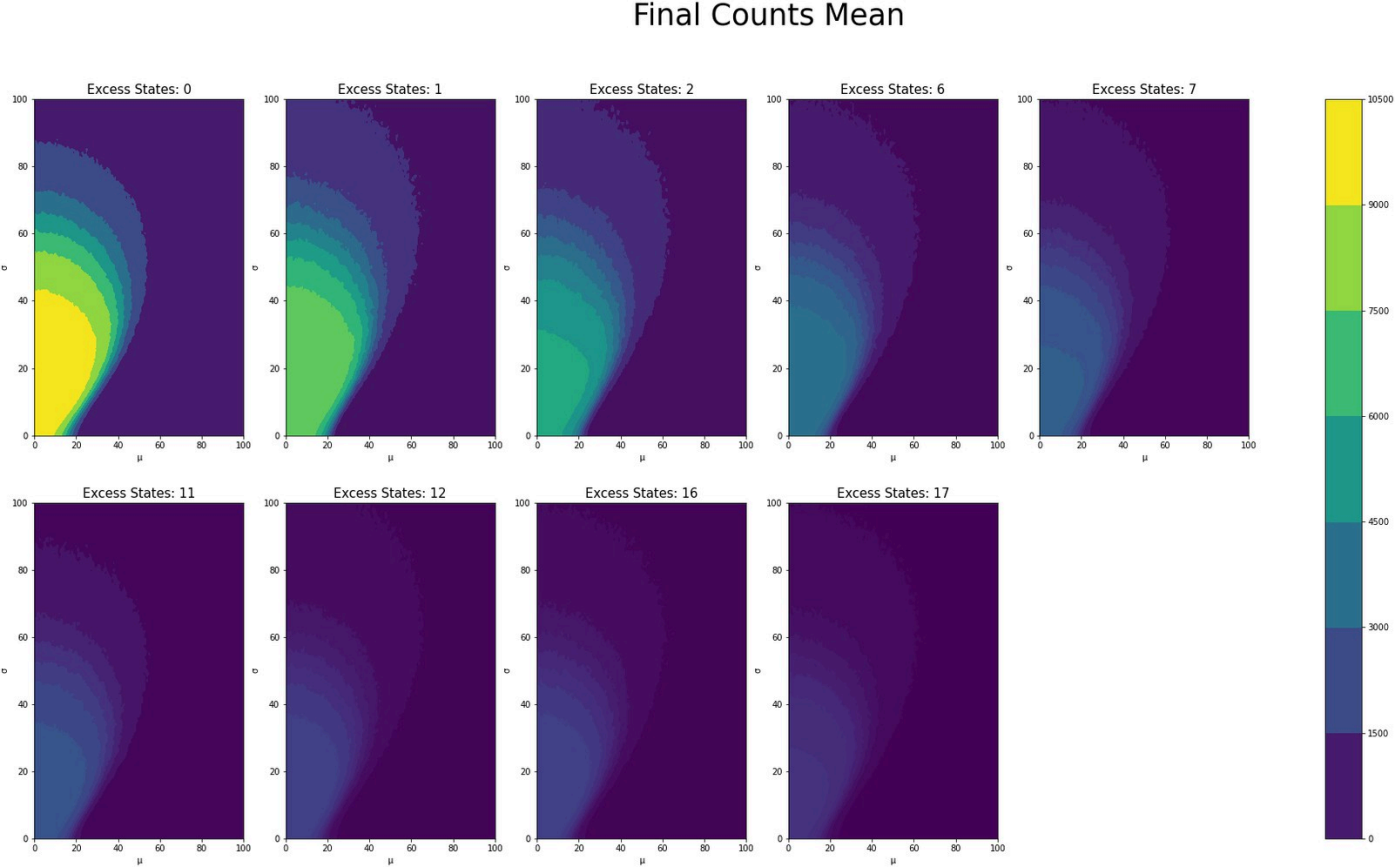


**Fig 3:**
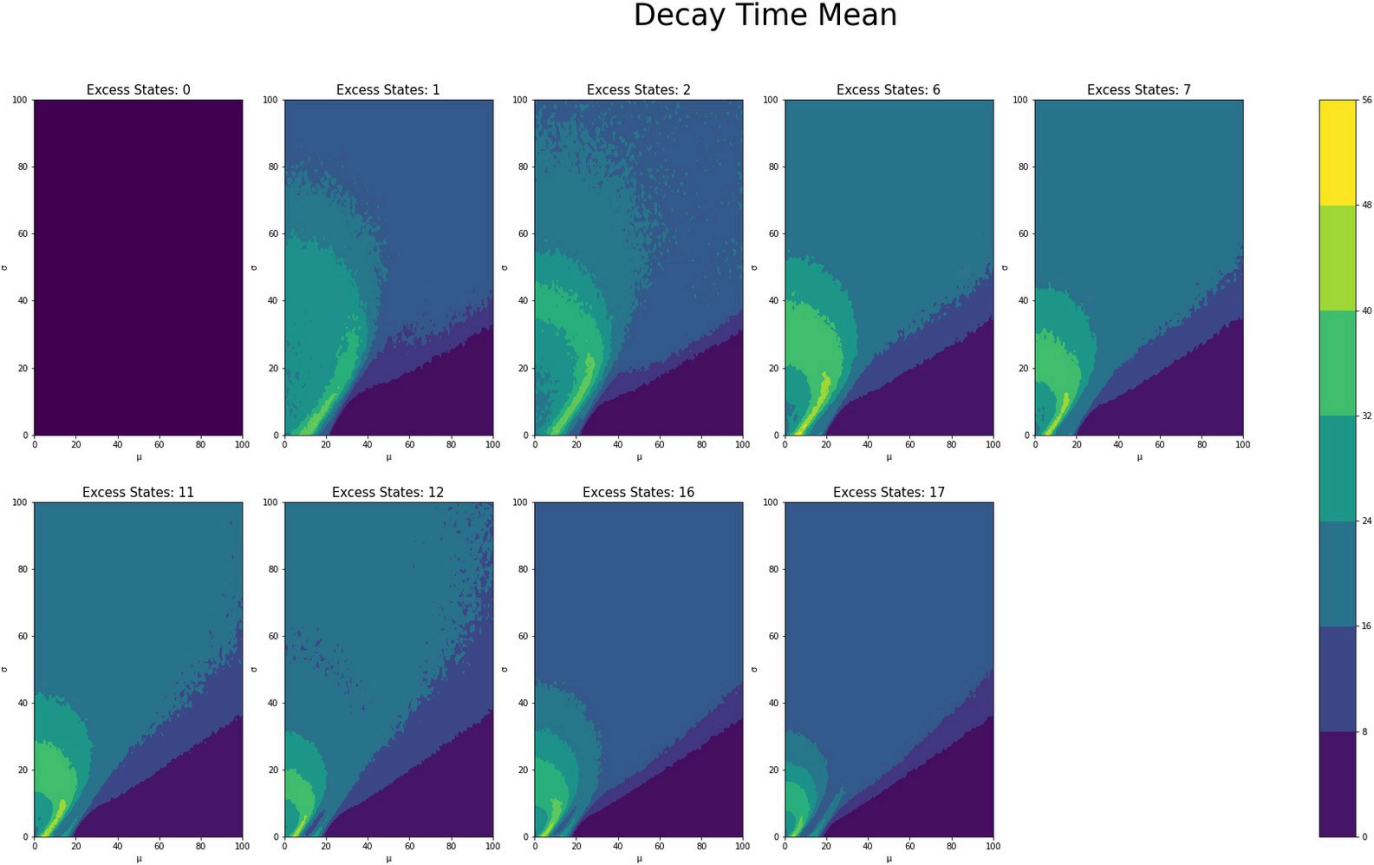


**Fig 4:**
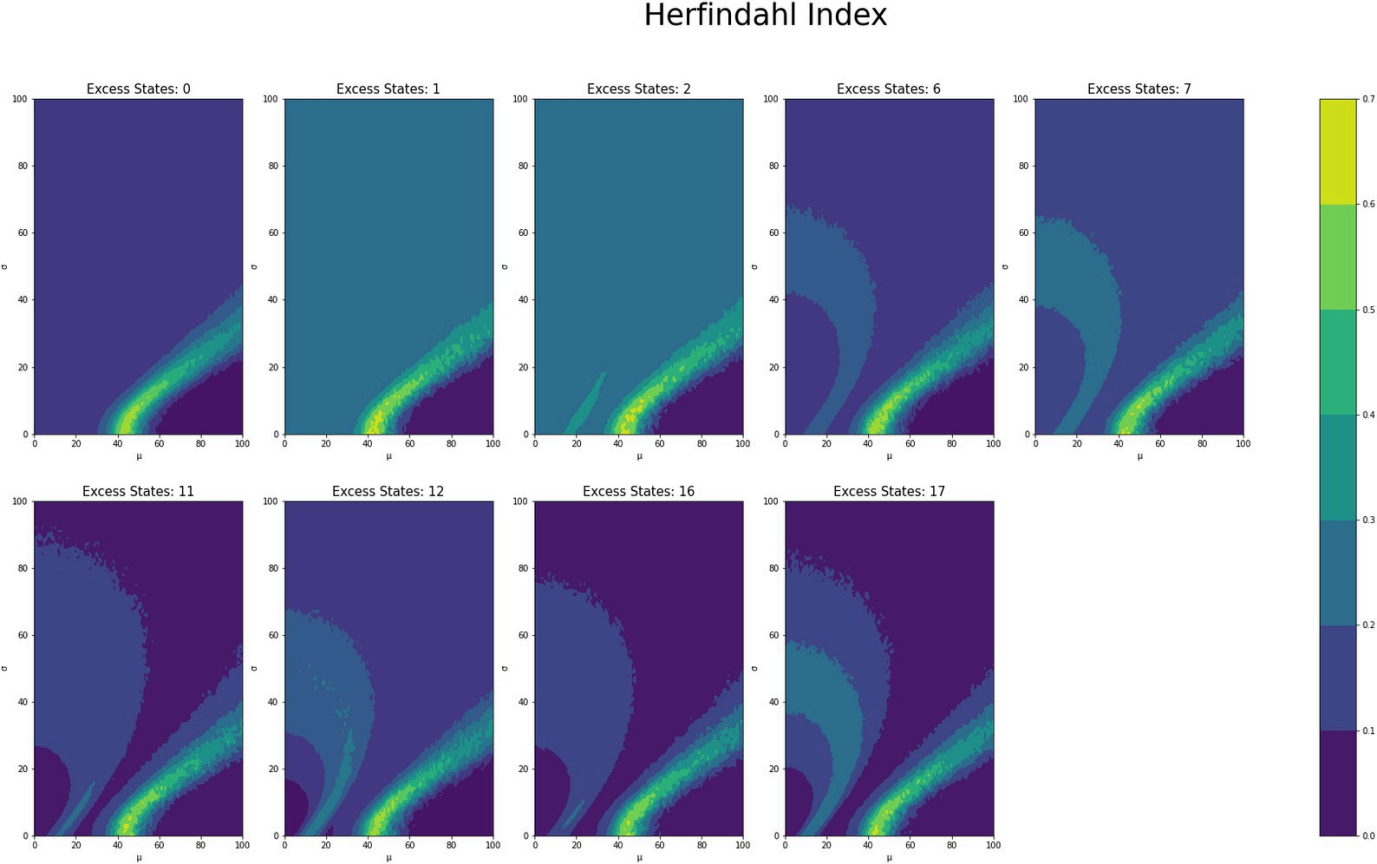


**Fig 5:**
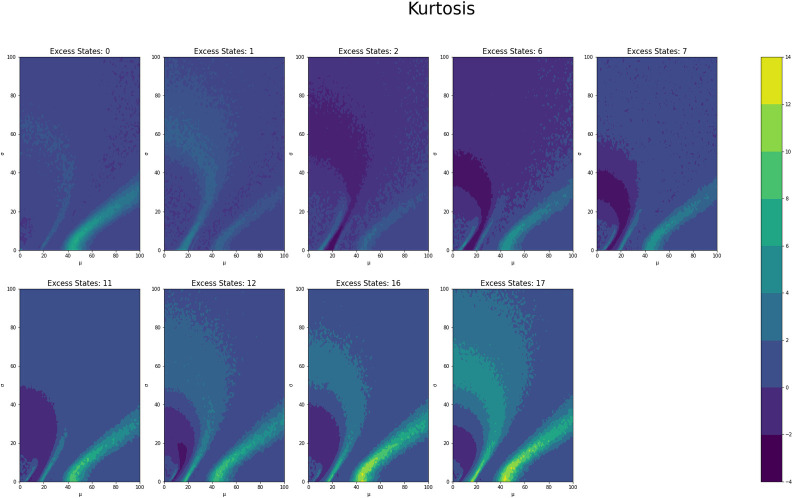


Fig 1 shows the average counts averaged across states for each *μ* and *σ* value grouped by number of excess states, while Figs 2 and 3 shows the same type of plot for final counts and decay time respectively. These plots have similar fan like shape across both excess states as well as the range of *μ* and *σ* combinations for which there are interesting count values. Figs 4 and 5 show the Herfindahl index and kurtosis respectively averaged by each *μ* and *σ* pair grouped by number of excess states. Given the similarity between these groups of graphs, it suggests that the Herfindahl index and kurtosis are both effectively functioning as measures of concentration across the states. Notably both the Herfindahl index and kurtosis reach high values in a band across the bottom right corner going from around *μ* = 40 to *σ* = 30.

**Fig 6:**
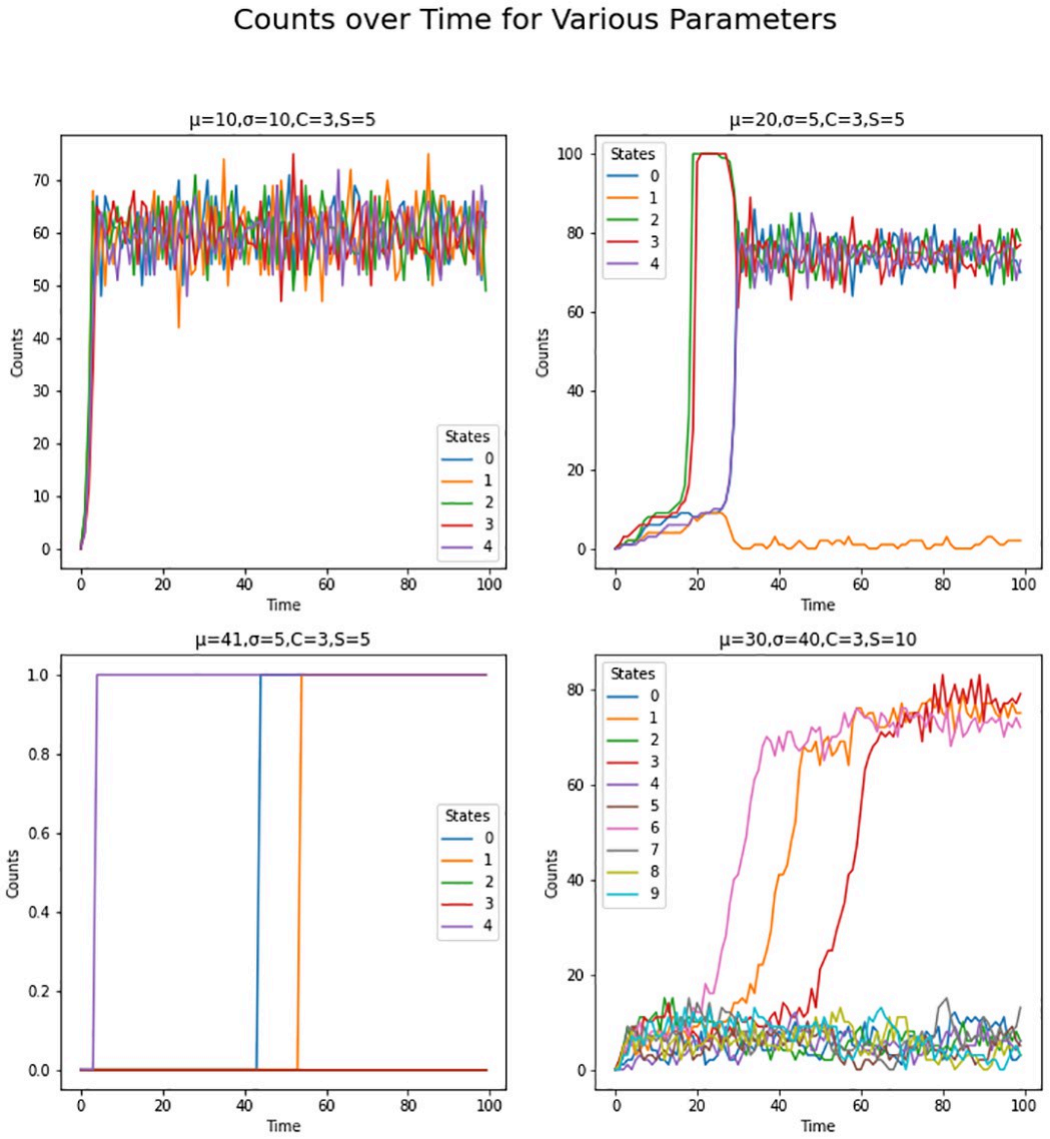


Fig 6 shows the counts over time from four different runs with different configurations of parameters. The lower left graph has *μ* = 41, *σ* = 5, and 2 excess states putting it within the high concentration band. There are not many total counts in this run, and since the high concentration bad overlaps with areas of low final and average counts generally this suggest that in this high concentration there is minimal adoption of any states. Thus this region of the parameter space somewhat resembles a natural monopoly, where it is difficult to successfully enter and those that do dominate.

### Regressions

The primary independent variable of interest is excess states, which is S-C. The OLS regressions follow the form: *Y* = *β*_1_*ExcessStates* + *β*_2_*μ* + *β*_3_*σ* + *c*. Tables 3 and 4 show the regression coefficients for the variable excess states for all outcomes of interest. Each time aggregation—state aggregation pair is the dependant variable. So, the mean across states of the average counts over time is a separate model from the one considering the range across states of the average counts over time. All coefficients listed in this table are statistically significant at the 1% level. In Table 3 the columns Mean, Standard Deviation, Max, and Range refer to the method of aggregation across states within a run.

**Table 3:** Excess states regression coefficient.

	State Aggregations			
Time Aggregations	Mean	Standard Deviation	Max	Range
Final Counts	-131.1854	18.9546	-75.6047	56.2781
Max Counts	-1.3529	0.3306	-0.5117	1.0086
Time to Max	-0.1939	0.6074	1.1336	2.1422
Average Counts	-1.3119	0.1895	-0.7560	0.5628
Variance in Counts	-7.5407	0.6448	-3.1413	2.4104
Min after Max	-1.5651	0.2450	-0.8403	0.7280
Decay Time	0.6567	0.6964	2.6354	2.6562
Entropy in Counts	-0.0261	0.0207	0.0128	0.0717

**Table 4:** Excess states regression coefficient for global variables.

	Coefficient
Herfindahl Index	-0.0019
Kurtosis in Counts	0.0779
Number of Zero Counts	0.0853

As a robustness check, I also preformed aggregations by Median and Mean Absolute Deviation. The Median is used as an outlier robust aggregation for the center of the distribution (compared to the Mean) and Mean Absolute Deviation is used as an outlier robust measure of dispersion (compared to Standard Deviation). The table with regression coefficients for Median and Mean Absolute Deviation aggregations are in the S1 File.

As the results in Table 3 shows the sign for excess states coefficient for all measures except decay time are negative. For decay time this suggests that increasing excess states increases time between the maximum and the minimum after the maximum. For the rest of the outcomes of interest this suggests that increasing the number of excess stats is associated with less overall reductions on average. The reductions in the dispersion measures of variance in counts and entropy in counts suggests that increasing excess states leads to runs which are more consistent in time. On the other hand a negative coefficient for Final Counts, Max Counts, Average Counts and Min after Max suggest less average adoption across states.

Alternatively the coefficient for the range aggregation has a positive coefficient for all of the outcomes of interest. With the exception of Time to Maximum and Decay Time, the coefficients for the maximum aggregation are negative. In these cases given the coefficients for the means, and the range this implies that while the increase in excess states reduces the average and maximum level, the impact disproportional effects the minimum since the range increases. When looking at the number of zeros, the regression coefficient for excess states is also positive, providing additional evidence for disproportionate effect of increasing excess states on the minimum counts. For Time to Maximum and Variance in Counts, it is possible that the positive effect of excess states on the range is due to the increases in the maximum. The broadly suggests that increasing the number of states can widen the disparities between the popularity of the states.

Similar to the range, the regression coefficients for the standard deviation aggregation are positive for all outcomes. [28] use standard deviation as a measure of unpredictability, but while they were measuring unpredictability across runs this measures unpredictability across states. This suggests that increasing the excess states makes all of the outcomes of interest less predictable.

The full regression tables are listed in the S1 File.

## Discussion

The parameter configurations shown in Fig 6 demonstrated four different competitive regimes. For example while both *μ* = 10, *σ* = 5, *C* = 3, *S* = 5 and *μ* = 20, *σ* = 5, *C* = 3, *S* = 5 are both highly competitive, there is a clear losing state in *μ* = 20, *σ* = 5, *C* = 3, *S* = 5. On the other hand *μ* = 30, *σ* = 40, *C* = 3, *S* = 10 is characterized by the emergence of a few clear winners with the rest of the states struggling to compete. As I mentioned earlier, in *μ* = 41, *σ* = 5, *C* = 3, *S* = 5 adoption is very difficult, so the three states that end up getting adopted are only adopted once. This suggests that the threshold parameters do capture a rich enough space of possible outcomes.

One way to think of these results is in terms of observed and unobserved cascades, in the sense that we are more likely to observe cascades that succeed, but not those that fizzle. In the context of music, consider that for every artist that becomes an “overnight success” there may be many others toiling away in obscurity. In this sense the given states that do not achieve popularity could be thought of counter factually as ones that could have. Each of the cascading states was facing the same distribution of thresholds as each other, with the only differences in their outcomes being due to chance. This shows that large disparities in popularity can occur even without and underlying differences in the “quality” of the cascading state, and purely arise from structure. Due to the trade off between the different states imposed by the capacity constraint, single cascade models and multicascade models without the capacity constraint will miss the effect unpopular states have on successful states. This also suggests that just because something is popular, that does not imply it is high quality.

Since the capacity constraint provides a mechanism for agents to regularly remove states, this can help determine their behavior as they fade away. The decay time results suggest that while on average increases excess states reduces the decay time, it actually increases the maximum decay time. This suggests that the in more competitive environments the most stable states are even more stable. A similar pattern is shown with time to maximum, suggesting that in more competitive environments even though the average peak is earlier, the maximum peak is delayed. It is possible that this could be due to a sort of lock in effect, where once a state is adopted by a certain threshold of agents there is a minimum level it can no longer dip below. The analysis of minimum post maximum, suggest that this may not be the case as adding excess states decreases the maximum minimum post maximum. If there is lock in, this suggests that increasing excess states reduces the floor that states are locked in above.

## Conclusion

These results suggest that the capacity constraint may play an important role in the diffusion dynamics of environments with multiple states that could potentially cascade. Increasing the number of total states in excess of capacity is associated with increased concentration of popularity, larger disparities between popular and unpopular states as well as greater unpredictability in which states will become popular, even while the popularity of a given state over time tended to become more predictable. Unsurprisingly increased competition from greater excess states tended to reduce average popularity overall, the heterogeneous impact suggests that capacity constraints may play a role in driving the superstar phenomena that [22] describes. Since each of the states begin equally preferable, this suggests a mechanism for how random chance and structure can drive popularity as opposed to underlying value. Thus, more empirical work is needed to measure people’s capacities as well as determine the influence of the capacity constraint on real systems.

## Supporting information

S1 File (PDF)
