# Supplementary material for: Cascades in capacity constrained agents
Source: PLoS One. 2023 Jan 20;18(1):e0280326. doi: 10.1371/journal.pone.0280326 (PMC9858083; doi:10.1371/journal.pone.0280326)
Supplement: S1 File — (PDF) [file pone.0280326.s001.pdf]

Supplementary Tables for Cascades in Capacity Constrained Agents

1 Regression Coefficients Table for Median and Mean Absolute Deviation 2

2 Excess States Regressions 4

2.1 Average Counts 4
2.2 Herfindahl Index 8
2.3 Kurtosis in Counts 9
2.4 Number of Zero Counts 10
2.5 Decay Time 11
2.6 Entropy in Counts 15
2.7 Final Counts 19
2.8 Max Counts 23
2.9 Time of Max 27
2.10 Min after Max 31
2.11 Variance in Counts 35

1 Regression Coefficients Table for Median and Mean Absolute Deviation

Table 1. Excess States Regression Coefficient

	Median	Mean Absolute Deviation
Final Counts	-149.4903	0.2646
Max Counts	-1.5992	0.2646
Time to Max	-0.3647	0.5338
Average Counts	-1.4949	0.1487
Variance in Counts	-9.1234	0.2874
Min after Max	-1.8090	-0.8403
Decay Time	0.4166	0.5381
Entropy in Counts	-0.0304	0.0172

2 Excess States Regressions

2.1 Average Counts

**Dep. Variable:**	avg_count_gap	**R-squared:**	0.236
**Model:**	OLS	**Adj. R-squared:**	0.236
**Method:**	Least Squares	**F-statistic:**	1.005e+06
**Date:**	Tue, 15 Nov 2022	**Prob (F-statistic):**	0.00
**Time:**	23:28:05	**Log-Likelihood:**	-5.1007e+07
**No. Observations:**	12241200	**AIC:**	1.020e+08
**Df Residuals:**	12241196	**BIC:**	1.020e+08
**Df Model:**	3		

	**coef**	**std err**	**z**	**P> |z|**	**[0.025**	**0.975]**
**const**	23.2599	0.015	1512.622	0.000	23.230	23.290
**mu**	-0.2679	0.000	-1676.313	0.000	-0.268	-0.268
**sigma**	-0.0482	0.000	-350.811	0.000	-0.049	-0.048
**excess_states**	0.5628	0.001	764.582	0.000	0.561	0.564

**Omnibus:**	4080493.816	**Durbin-Watson:**	0.631
**Prob(Omnibus):**	0.000	**Jarque-Bera (JB):**	12513048.042
**Skew:**	1.750	**Prob(JB):**	0.00
**Kurtosis:**	6.504	**Cond. No.**	214.

Table 2. Average Counts Range

**Dep. Variable:**	avg_count_mad	**R-squared:**	0.202
**Model:**	OLS	**Adj. R-squared:**	0.202
**Method:**	Least Squares	**F-statistic:**	8.198e+05
**Date:**	Tue, 15 Nov 2022	**Prob (F-statistic):**	0.00
**Time:**	23:28:03	**Log-Likelihood:**	-3.8039e+07
**No. Observations:**	12241200	**AIC:**	7.608e+07
**Df Residuals:**	12241196	**BIC:**	7.608e+07
**Df Model:**	3		

	**coef**	**std err**	**z**	**P> |z|**	**[0.025**	**0.975]**
**const**	7.6248	0.006	1375.010	0.000	7.614	7.636
**mu**	-0.0853	5.64e-05	-1514.236	0.000	-0.085	-0.085
**sigma**	-0.0208	4.74e-05	-438.688	0.000	-0.021	-0.021
**excess_states**	0.1487	0.000	668.951	0.000	0.148	0.149

**Omnibus:**	5777301.868	**Durbin-Watson:**	0.590
**Prob(Omnibus):**	0.000	**Jarque-Bera (JB):**	32445119.926
**Skew:**	2.268	**Prob(JB):**	0.00
**Kurtosis:**	9.560	**Cond. No.**	214.

Table 3. Average Counts Mean Absolute Deviation

**Dep. Variable:**	avg_count_max	**R-squared:**	0.458
**Model:**	OLS	**Adj. R-squared:**	0.458
**Method:**	Least Squares	**F-statistic:**	2.874e+06
**Date:**	Tue, 15 Nov 2022	**Prob (F-statistic):**	0.00
**Time:**	23:28:04	**Log-Likelihood:**	-5.4271e+07
**No. Observations:**	12241200	**AIC:**	1.085e+08
**Df Residuals:**	12241196	**BIC:**	1.085e+08
**Df Model:**	3		

	**coef**	**std err**	**z**	**P> |z|**	**[0.025**	**0.975]**
**const**	64.0276	0.022	2886.532	0.000	63.984	64.071
**mu**	-0.5982	0.000	-2920.922	0.000	-0.599	-0.598
**sigma**	-0.1719	0.000	-840.151	0.000	-0.172	-0.172
**excess_states**	-0.7560	0.001	-813.940	0.000	-0.758	-0.754

**Omnibus:**	501005.454	**Durbin-Watson:**	0.409
**Prob(Omnibus):**	0.000	**Jarque-Bera (JB):**	562347.338
**Skew:**	0.521	**Prob(JB):**	0.00
**Kurtosis:**	2.878	**Cond. No.**	214.

Table 4. Average Counts Max

**Dep. Variable:**	avg_count_mean	**R-squared:**	0.421
**Model:**	OLS	**Adj. R-squared:**	0.421
**Method:**	Least Squares	**F-statistic:**	1.618e+06
**Date:**	Tue, 15 Nov 2022	**Prob (F-statistic):**	0.00
**Time:**	23:28:00	**Log-Likelihood:**	-5.2822e+07
**No. Observations:**	12241200	**AIC:**	1.056e+08
**Df Residuals:**	12241196	**BIC:**	1.056e+08
**Df Model:**	3		

	**coef**	**std err**	**z**	**P> |z|**	**[0.025**	**0.975]**
**const**	50.4170	0.023	2212.697	0.000	50.372	50.462
**mu**	-0.4272	0.000	-2195.905	0.000	-0.428	-0.427
**sigma**	-0.1390	0.000	-787.681	0.000	-0.139	-0.139
**excess_states**	-1.3119	0.001	-1643.201	0.000	-1.313	-1.310

**Omnibus:**	2584744.069	**Durbin-Watson:**	0.280
**Prob(Omnibus):**	0.000	**Jarque-Bera (JB):**	5154832.357
**Skew:**	1.274	**Prob(JB):**	0.00
**Kurtosis:**	4.900	**Cond. No.**	214.

Table 5. Average Counts Mean

**Dep. Variable:**	avg_count_median	**R-squared:**	0.395
**Model:**	OLS	**Adj. R-squared:**	0.395
**Method:**	Least Squares	**F-statistic:**	1.424e+06
**Date:**	Tue, 15 Nov 2022	**Prob (F-statistic):**	0.00
**Time:**	23:28:01	**Log-Likelihood:**	-5.3409e+07
**No. Observations:**	12241200	**AIC:**	1.068e+08
**Df Residuals:**	12241196	**BIC:**	1.068e+08
**Df Model:**	3		

	**coef**	**std err**	**z**	**P> |z|**	**[0.025**	**0.975]**
**const**	48.5528	0.024	2036.872	0.000	48.506	48.600
**mu**	-0.3970	0.000	-1938.050	0.000	-0.397	-0.397
**sigma**	-0.1285	0.000	-710.698	0.000	-0.129	-0.128
**excess_states**	-1.4949	0.001	-1786.790	0.000	-1.497	-1.493

**Omnibus:**	2442547.775	**Durbin-Watson:**	0.299
**Prob(Omnibus):**	0.000	**Jarque-Bera (JB):**	4488472.873
**Skew:**	1.259	**Prob(JB):**	0.00
**Kurtosis:**	4.569	**Cond. No.**	214.

Table 6. Average Counts Median

**Dep. Variable:**	avg_count_std	**R-squared:**	0.216
**Model:**	OLS	**Adj. R-squared:**	0.216
**Method:**	Least Squares	**F-statistic:**	8.800e+05
**Date:**	Tue, 15 Nov 2022	**Prob (F-statistic):**	0.00
**Time:**	23:28:02	**Log-Likelihood:**	-3.9432e+07
**No. Observations:**	12241200	**AIC:**	7.886e+07
**Df Residuals:**	12241196	**BIC:**	7.886e+07
**Df Model:**	3		

	**coef**	**std err**	**z**	**P> |z|**	**[0.025**	**0.975]**
**const**	8.7249	0.006	1428.903	0.000	8.713	8.737
**mu**	-0.0989	6.28e-05	-1574.523	0.000	-0.099	-0.099
**sigma**	-0.0227	5.33e-05	-426.064	0.000	-0.023	-0.023
**excess_states**	0.1895	0.000	713.127	0.000	0.189	0.190

**Omnibus:**	4992192.695	**Durbin-Watson:**	0.595
**Prob(Omnibus):**	0.000	**Jarque-Bera (JB):**	21062301.157
**Skew:**	2.024	**Prob(JB):**	0.00
**Kurtosis:**	7.991	**Cond. No.**	214.

Table 7. Average Counts Standard Deviation

2.2 Herfindahl Index

**Dep. Variable:**	counts_herfindahl_index	**R-squared:**	0.020
**Model:**	OLS	**Adj. R-squared:**	0.020
**Method:**	Least Squares	**F-statistic:**	1.092e+05
**Date:**	Tue, 15 Nov 2022	**Prob (F-statistic):**	0.00
**Time:**	22:55:11	**Log-Likelihood:**	6.5730e+06
**No. Observations:**	12241200	**AIC:**	-1.315e+07
**Df Residuals:**	12241196	**BIC:**	-1.315e+07
**Df Model:**	3		

	**coef**	**std err**	**z**	**P> |z|**	**[0.025**	**0.975]**
**const**	0.1935	0.000	1772.484	0.000	0.193	0.194
**mu**	-0.0002	1.29e-06	-178.240	0.000	-0.000	-0.000
**sigma**	-0.0005	1.66e-06	-318.575	0.000	-0.001	-0.001
**excess_states**	-0.0019	6.44e-06	-289.926	0.000	-0.002	-0.002

**Omnibus:**	9554483.763	**Durbin-Watson:**	1.235
**Prob(Omnibus):**	0.000	**Jarque-Bera (JB):**	210562976.489
**Skew:**	3.683	**Prob(JB):**	0.00
**Kurtosis:**	21.936	**Cond. No.**	214.

Table 8. Herfindahl Index

2.3 Kurtosis in Counts

**Dep. Variable:**	counts_kurtosis	**R-squared:**	0.034
**Model:**	OLS	**Adj. R-squared:**	0.034
**Method:**	Least Squares	**F-statistic:**	9.759e+04
**Date:**	Tue, 15 Nov 2022	**Prob (F-statistic):**	0.00
**Time:**	22:55:09	**Log-Likelihood:**	-3.0424e+07
**No. Observations:**	12241200	**AIC:**	6.085e+07
**Df Residuals:**	12241196	**BIC:**	6.085e+07
**Df Model:**	3		

	**coef**	**std err**	**z**	**P> |z|**	**[0.025**	**0.975]**
**const**	0.5543	0.002	242.380	0.000	0.550	0.559
**mu**	0.0019	2.67e-05	71.537	0.000	0.002	0.002
**sigma**	-0.0081	2.98e-05	-270.933	0.000	-0.008	-0.008
**excess_states**	0.0779	0.000	504.062	0.000	0.078	0.078

**Omnibus:**	7396858.354	**Durbin-Watson:**	1.593
**Prob(Omnibus):**	0.000	**Jarque-Bera (JB):**	83304645.886
**Skew:**	2.774	**Prob(JB):**	0.00
**Kurtosis:**	14.513	**Cond. No.**	214.

Table 9. Kurtosis in Counts

2.4 Number of Zero Counts

**Dep. Variable:**	counts_zeros	**R-squared:**	0.359
**Model:**	OLS	**Adj. R-squared:**	0.359
**Method:**	Least Squares	**F-statistic:**	1.133e+06
**Date:**	Tue, 15 Nov 2022	**Prob (F-statistic):**	0.00
**Time:**	22:55:06	**Log-Likelihood:**	-3.3351e+07
**No. Observations:**	12241200	**AIC:**	6.670e+07
**Df Residuals:**	12241196	**BIC:**	6.670e+07
**Df Model:**	3		

	**coef**	**std err**	**z**	**P> |z|**	**[0.025**	**0.975]**
**const**	2.4025	0.003	953.627	0.000	2.398	2.407
**mu**	0.0533	3.76e-05	1416.991	0.000	0.053	0.053
**sigma**	-0.0763	4.36e-05	-1748.111	0.000	-0.076	-0.076
**excess_states**	0.0853	0.000	420.039	0.000	0.085	0.086

**Omnibus:**	3968692.032	**Durbin-Watson:**	0.231
**Prob(Omnibus):**	0.000	**Jarque-Bera (JB):**	12174137.117
**Skew:**	1.696	**Prob(JB):**	0.00
**Kurtosis:**	6.515	**Cond. No.**	214.

Table 10. Number of Zero Counts

2.5 Decay Time

**Dep. Variable:**	decay_time_gap	**R-squared:**	0.415
**Model:**	OLS	**Adj. R-squared:**	0.415
**Method:**	Least Squares	**F-statistic:**	4.668e+06
**Date:**	Tue, 15 Nov 2022	**Prob (F-statistic):**	0.00
**Time:**	23:36:14	**Log-Likelihood:**	-5.5133e+07
**No. Observations:**	12241200	**AIC:**	1.103e+08
**Df Residuals:**	12241196	**BIC:**	1.103e+08
**Df Model:**	3		

	**coef**	**std err**	**z**	**P>|z|**	**[0.025**	**0.975]**
**const**	13.3829	0.018	727.188	0.000	13.347	13.419
**mu**	-0.2004	0.000	-895.526	0.000	-0.201	-0.200
**sigma**	0.1889	0.000	864.407	0.000	0.188	0.189
**excess_states**	2.6562	0.001	2697.139	0.000	2.654	2.658

**Omnibus:**	470389.766	**Durbin-Watson:**	0.694
**Prob(Omnibus):**	0.000	**Jarque-Bera (JB):**	527117.409
**Skew:**	0.508	**Prob(JB):**	0.00
**Kurtosis:**	3.023	**Cond. No.**	214.

Table 11. Decay Time Range

**Dep. Variable:**	decay_time_mad	**R-squared:**	0.280
**Model:**	OLS	**Adj. R-squared:**	0.280
**Method:**	Least Squares	**F-statistic:**	2.766e+06
**Date:**	Tue, 15 Nov 2022	**Prob (F-statistic):**	0.00
**Time:**	23:36:12	**Log-Likelihood:**	-3.9998e+07
**No. Observations:**	12241200	**AIC:**	8.000e+07
**Df Residuals:**	12241196	**BIC:**	8.000e+07
**Df Model:**	3		

	**coef**	**std err**	**z**	**P>|z|**	**[0.025**	**0.975]**
**const**	4.5782	0.006	809.974	0.000	4.567	4.589
**mu**	-0.0549	6.55e-05	-837.584	0.000	-0.055	-0.055
**sigma**	0.0466	6.17e-05	754.067	0.000	0.046	0.047
**excess_states**	0.5381	0.000	2046.561	0.000	0.538	0.539

**Omnibus:**	1611219.795	**Durbin-Watson:**	0.649
**Prob(Omnibus):**	0.000	**Jarque-Bera (JB):**	2372560.760
**Skew:**	0.984	**Prob(JB):**	0.00
**Kurtosis:**	3.881	**Cond. No.**	214.

Table 12. Decay Time Mean Absolute Deviation

**Dep. Variable:**	decay_time_max	**R-squared:**	0.403
**Model:**	OLS	**Adj. R-squared:**	0.403
**Method:**	Least Squares	**F-statistic:**	4.487e+06
**Date:**	Tue, 15 Nov 2022	**Prob (F-statistic):**	0.00
**Time:**	23:36:13	**Log-Likelihood:**	-5.5408e+07
**No. Observations:**	12241200	**AIC:**	1.108e+08
**Df Residuals:**	12241196	**BIC:**	1.108e+08
**Df Model:**	3		

	**coef**	**std err**	**z**	**P> |z|**	**[0.025**	**0.975]**
**const**	15.4832	0.019	807.895	0.000	15.446	15.521
**mu**	-0.2099	0.000	-915.425	0.000	-0.210	-0.209
**sigma**	0.1847	0.000	828.469	0.000	0.184	0.185
**excess_states**	2.6354	0.001	2638.007	0.000	2.633	2.637

**Omnibus:**	510816.452	**Durbin-Watson:**	0.666
**Prob(Omnibus):**	0.000	**Jarque-Bera (JB):**	578052.821
**Skew:**	0.532	**Prob(JB):**	0.00
**Kurtosis:**	3.001	**Cond. No.**	214.

Table 13. Decay Time Max

**Dep. Variable:**	decay_time_mean	**R-squared:**	0.251
**Model:**	OLS	**Adj. R-squared:**	0.251
**Method:**	Least Squares	**F-statistic:**	2.552e+06
**Date:**	Tue, 15 Nov 2022	**Prob (F-statistic):**	0.00
**Time:**	23:36:09	**Log-Likelihood:**	-4.4100e+07
**No. Observations:**	12241200	**AIC:**	8.820e+07
**Df Residuals:**	12241196	**BIC:**	8.820e+07
**Df Model:**	3		

	**coef**	**std err**	**z**	**P> |z|**	**[0.025**	**0.975]**
**const**	8.7443	0.009	997.983	0.000	8.727	8.761
**mu**	-0.0926	9.37e-05	-987.826	0.000	-0.093	-0.092
**sigma**	0.0515	8.63e-05	596.540	0.000	0.051	0.052
**excess_states**	0.6567	0.000	1841.311	0.000	0.656	0.657

**Omnibus:**	2729531.590	**Durbin-Watson:**	0.568
**Prob(Omnibus):**	0.000	**Jarque-Bera (JB):**	6072691.552
**Skew:**	1.282	**Prob(JB):**	0.00
**Kurtosis:**	5.309	**Cond. No.**	214.

Table 14. Decay Time Mean

**Dep. Variable:**	decay_time_median	**R-squared:**	0.157
**Model:**	OLS	**Adj. R-squared:**	0.157
**Method:**	Least Squares	**F-statistic:**	1.389e+06
**Date:**	Tue, 15 Nov 2022	**Prob (F-statistic):**	0.00
**Time:**	23:36:10	**Log-Likelihood:**	-4.3430e+07
**No. Observations:**	12241200	**AIC:**	8.686e+07
**Df Residuals:**	12241196	**BIC:**	8.686e+07
**Df Model:**	3		

	**coef**	**std err**	**z**	**P> |z|**	**[0.025**	**0.975]**
**const**	7.9233	0.009	913.565	0.000	7.906	7.940
**mu**	-0.0809	8.95e-05	-903.362	0.000	-0.081	-0.081
**sigma**	0.0319	8.02e-05	397.214	0.000	0.032	0.032
**excess_states**	0.4166	0.000	1289.265	0.000	0.416	0.417

**Omnibus:**	5901304.944	**Durbin-Watson:**	0.912
**Prob(Omnibus):**	0.000	**Jarque-Bera (JB):**	40558448.479
**Skew:**	2.238	**Prob(JB):**	0.00
**Kurtosis:**	10.713	**Cond. No.**	214.

Table 15. Decay Time Median

**Dep. Variable:**	decay_time_std	**R-squared:**	0.313
**Model:**	OLS	**Adj. R-squared:**	0.313
**Method:**	Least Squares	**F-statistic:**	3.229e+06
**Date:**	Tue, 15 Nov 2022	**Prob (F-statistic):**	0.00
**Time:**	23:36:11	**Log-Likelihood:**	-4.1938e+07
**No. Observations:**	12241200	**AIC:**	8.388e+07
**Df Residuals:**	12241196	**BIC:**	8.388e+07
**Df Model:**	3		

	**coef**	**std err**	**z**	**P> |z|**	**[0.025**	**0.975]**
**const**	5.1992	0.006	800.173	0.000	5.186	5.212
**mu**	-0.0645	7.64e-05	-844.423	0.000	-0.065	-0.064
**sigma**	0.0575	7.27e-05	790.293	0.000	0.057	0.058
**excess_states**	0.6964	0.000	2224.352	0.000	0.696	0.697

**Omnibus:**	1174903.619	**Durbin-Watson:**	0.644
**Prob(Omnibus):**	0.000	**Jarque-Bera (JB):**	1543226.865
**Skew:**	0.837	**Prob(JB):**	0.00
**Kurtosis:**	3.470	**Cond. No.**	214.

Table 16. Decay Time Standard Deviation

2.6 Entropy in Counts

**Dep. Variable:**	entropy_gap	**R-squared:**	0.490
**Model:**	OLS	**Adj. R-squared:**	0.490
**Method:**	Least Squares	**F-statistic:**	2.915e+06
**Date:**	Tue, 15 Nov 2022	**Prob (F-statistic):**	0.00
**Time:**	23:36:36	**Log-Likelihood:**	-6.3190e+06
**No. Observations:**	10061128	**AIC:**	1.264e+07
**Df Residuals:**	10061124	**BIC:**	1.264e+07
**Df Model:**	3		

	**coef**	**std err**	**z**	**P> |z|**	**[0.025**	**0.975]**
**const**	0.5309	0.000	1222.713	0.000	0.530	0.532
**mu**	-0.0004	5.11e-06	-70.739	0.000	-0.000	-0.000
**sigma**	0.0005	5.72e-06	91.385	0.000	0.001	0.001
**excess_states**	0.0717	2.46e-05	2914.515	0.000	0.072	0.072

**Omnibus:**	424113.884	**Durbin-Watson:**	1.149
**Prob(Omnibus):**	0.000	**Jarque-Bera (JB):**	482576.425
**Skew:**	0.513	**Prob(JB):**	0.00
**Kurtosis:**	3.312	**Cond. No.**	220.

Table 17. Entropy in Counts Range

**Dep. Variable:**	entropy_mad	**R-squared:**	0.361
**Model:**	OLS	**Adj. R-squared:**	0.361
**Method:**	Least Squares	**F-statistic:**	1.817e+06
**Date:**	Tue, 15 Nov 2022	**Prob (F-statistic):**	0.00
**Time:**	23:36:31	**Log-Likelihood:**	5.2894e+06
**No. Observations:**	10061128	**AIC:**	-1.058e+07
**Df Residuals:**	10061124	**BIC:**	-1.058e+07
**Df Model:**	3		

	**coef**	**std err**	**z**	**P> |z|**	**[0.025**	**0.975]**
**const**	0.1845	0.000	1287.414	0.000	0.184	0.185
**mu**	-0.0004	1.54e-06	-262.975	0.000	-0.000	-0.000
**sigma**	-0.0003	1.78e-06	-140.640	0.000	-0.000	-0.000
**excess_states**	0.0172	7.45e-06	2310.460	0.000	0.017	0.017

**Omnibus:**	1753048.006	**Durbin-Watson:**	1.094
**Prob(Omnibus):**	0.000	**Jarque-Bera (JB):**	3553133.826
**Skew:**	1.054	**Prob(JB):**	0.00
**Kurtosis:**	5.009	**Cond. No.**	220.

Table 18. Entropy in Counts Mean Absolute Deviation

**Dep. Variable:**	entropy_max	**R-squared:**	0.291
**Model:**	OLS	**Adj. R-squared:**	0.291
**Method:**	Least Squares	**F-statistic:**	1.455e+06
**Date:**	Tue, 15 Nov 2022	**Prob (F-statistic):**	0.00
**Time:**	23:36:33	**Log-Likelihood:**	-7.8941e+06
**No. Observations:**	10061128	**AIC:**	1.579e+07
**Df Residuals:**	10061124	**BIC:**	1.579e+07
**Df Model:**	3		

	**coef**	**std err**	**z**	**P> |z|**	**[0.025**	**0.975]**
**const**	2.7206	0.001	4185.021	0.000	2.719	2.722
**mu**	-0.0119	6.1e-06	-1953.594	0.000	-0.012	-0.012
**sigma**	0.0021	7.44e-06	282.445	0.000	0.002	0.002
**excess_states**	0.0128	2.69e-05	474.969	0.000	0.013	0.013

**Omnibus:**	528581.054	**Durbin-Watson:**	0.565
**Prob(Omnibus):**	0.000	**Jarque-Bera (JB):**	641035.033
**Skew:**	-0.552	**Prob(JB):**	0.00
**Kurtosis:**	3.559	**Cond. No.**	220.

Table 19. Entropy in Counts Max

**Dep. Variable:**	entropy_mean	**R-squared:**	0.326
**Model:**	OLS	**Adj. R-squared:**	0.326
**Method:**	Least Squares	**F-statistic:**	1.612e+06
**Date:**	Tue, 15 Nov 2022	**Prob (F-statistic):**	0.00
**Time:**	23:36:25	**Log-Likelihood:**	-7.2175e+06
**No. Observations:**	10061128	**AIC:**	1.443e+07
**Df Residuals:**	10061124	**BIC:**	1.444e+07
**Df Model:**	3		

	**coef**	**std err**	**z**	**P> |z|**	**[0.025**	**0.975]**
**const**	2.4521	0.001	3858.037	0.000	2.451	2.453
**mu**	-0.0112	5.86e-06	-1907.969	0.000	-0.011	-0.011
**sigma**	0.0021	7.34e-06	284.985	0.000	0.002	0.002
**excess_states**	-0.0261	2.32e-05	-1125.613	0.000	-0.026	-0.026

**Omnibus:**	422874.215	**Durbin-Watson:**	0.498
**Prob(Omnibus):**	0.000	**Jarque-Bera (JB):**	559751.669
**Skew:**	-0.436	**Prob(JB):**	0.00
**Kurtosis:**	3.757	**Cond. No.**	220.

Table 20. Entropy in Counts Mean

**Dep. Variable:**	entropy_median	**R-squared:**	0.306
**Model:**	OLS	**Adj. R-squared:**	0.306
**Method:**	Least Squares	**F-statistic:**	1.569e+06
**Date:**	Tue, 15 Nov 2022	**Prob (F-statistic):**	0.00
**Time:**	23:36:27	**Log-Likelihood:**	-7.6622e+06
**No. Observations:**	10061128	**AIC:**	1.532e+07
**Df Residuals:**	10061124	**BIC:**	1.532e+07
**Df Model:**	3		

	**coef**	**std err**	**z**	**P> |z|**	**[0.025**	**0.975]**
**const**	2.4356	0.001	3718.088	0.000	2.434	2.437
**mu**	-0.0106	6.11e-06	-1738.678	0.000	-0.011	-0.011
**sigma**	0.0023	7.56e-06	308.465	0.000	0.002	0.002
**excess_states**	-0.0304	2.42e-05	-1255.078	0.000	-0.030	-0.030

**Omnibus:**	248228.266	**Durbin-Watson:**	0.574
**Prob(Omnibus):**	0.000	**Jarque-Bera (JB):**	376316.877
**Skew:**	-0.259	**Prob(JB):**	0.00
**Kurtosis:**	3.793	**Cond. No.**	220.

Table 21. Entropy in Counts Median

**Dep. Variable:**	entropy_std	**R-squared:**	0.404
**Model:**	OLS	**Adj. R-squared:**	0.404
**Method:**	Least Squares	**F-statistic:**	2.129e+06
**Date:**	Tue, 15 Nov 2022	**Prob (F-statistic):**	0.00
**Time:**	23:36:29	**Log-Likelihood:**	4.3740e+06
**No. Observations:**	10061128	**AIC:**	-8.748e+06
**Df Residuals:**	10061124	**BIC:**	-8.748e+06
**Df Model:**	3		

	**coef**	**std err**	**z**	**P> |z|**	**[0.025**	**0.975]**
**const**	0.2068	0.000	1321.313	0.000	0.207	0.207
**mu**	-0.0003	1.71e-06	-202.862	0.000	-0.000	-0.000
**sigma**	-0.0002	1.96e-06	-96.266	0.000	-0.000	-0.000
**excess_states**	0.0207	8.29e-06	2500.491	0.000	0.021	0.021

**Omnibus:**	1054508.326	**Durbin-Watson:**	1.089
**Prob(Omnibus):**	0.000	**Jarque-Bera (JB):**	1576171.688
**Skew:**	0.793	**Prob(JB):**	0.00
**Kurtosis:**	4.115	**Cond. No.**	220.

Table 22. Entropy in Counts Standard Deviation

2.7 Final Counts

**Dep. Variable:**	final_counts_sums_gap	**R-squared:**	0.236
**Model:**	OLS	**Adj. R-squared:**	0.236
**Method:**	Least Squares	**F-statistic:**	1.005e+06
**Date:**	Tue, 15 Nov 2022	**Prob (F-statistic):**	0.00
**Time:**	22:55:03	**Log-Likelihood:**	-1.0738e+08
**No. Observations:**	12241200	**AIC:**	2.148e+08
**Df Residuals:**	12241196	**BIC:**	2.148e+08
**Df Model:**	3		

	**coef**	**std err**	**z**	**P>|z|**	**[0.025**	**0.975]**
**const**	2325.9872	1.538	1512.622	0.000	2322.973	2329.001
**mu**	-26.7879	0.016	-1676.313	0.000	-26.819	-26.757
**sigma**	-4.8238	0.014	-350.811	0.000	-4.851	-4.797
**excess_states**	56.2781	0.074	764.582	0.000	56.134	56.422

**Omnibus:**	4080493.816	**Durbin-Watson:**	0.631
**Prob(Omnibus):**	0.000	**Jarque-Bera (JB):**	12513048.042
**Skew:**	1.750	**Prob(JB):**	0.00
**Kurtosis:**	6.504	**Cond. No.**	214.

Table 23. Final Counts Range

**Dep. Variable:**	final_counts_sums_mad	**R-squared:**	0.202
**Model:**	OLS	**Adj. R-squared:**	0.202
**Method:**	Least Squares	**F-statistic:**	8.198e+05
**Date:**	Tue, 15 Nov 2022	**Prob (F-statistic):**	0.00
**Time:**	22:55:02	**Log-Likelihood:**	-9.4411e+07
**No. Observations:**	12241200	**AIC:**	1.888e+08
**Df Residuals:**	12241196	**BIC:**	1.888e+08
**Df Model:**	3		

	**coef**	**std err**	**z**	**P>|z|**	**[0.025**	**0.975]**
**const**	762.4803	0.555	1375.010	0.000	761.393	763.567
**mu**	-8.5336	0.006	-1514.236	0.000	-8.545	-8.523
**sigma**	-2.0780	0.005	-438.688	0.000	-2.087	-2.069
**excess_states**	14.8734	0.022	668.951	0.000	14.830	14.917

**Omnibus:**	5777301.868	**Durbin-Watson:**	0.590
**Prob(Omnibus):**	0.000	**Jarque-Bera (JB):**	32445119.926
**Skew:**	2.268	**Prob(JB):**	0.00
**Kurtosis:**	9.560	**Cond. No.**	214.

Table 24. Final Counts Mean Absolute Deviation

**Dep. Variable:**	final_counts_sums_max	**R-squared:**	0.458
**Model:**	OLS	**Adj. R-squared:**	0.458
**Method:**	Least Squares	**F-statistic:**	2.874e+06
**Date:**	Tue, 15 Nov 2022	**Prob (F-statistic):**	0.00
**Time:**	22:55:02	**Log-Likelihood:**	-1.1064e+08
**No. Observations:**	12241200	**AIC:**	2.213e+08
**Df Residuals:**	12241196	**BIC:**	2.213e+08
**Df Model:**	3		

	**coef**	**std err**	**z**	**P> |z|**	**[0.025**	**0.975]**
**const**	6402.7643	2.218	2886.532	0.000	6398.417	6407.112
**mu**	-59.8240	0.020	-2920.922	0.000	-59.864	-59.784
**sigma**	-17.1927	0.020	-840.151	0.000	-17.233	-17.153
**excess_states**	-75.6047	0.093	-813.940	0.000	-75.787	-75.423

**Omnibus:**	501005.454	**Durbin-Watson:**	0.409
**Prob(Omnibus):**	0.000	**Jarque-Bera (JB):**	562347.338
**Skew:**	0.521	**Prob(JB):**	0.00
**Kurtosis:**	2.878	**Cond. No.**	214.

Table 25. Final Counts Max

**Dep. Variable:**	final_counts_sums_mean	**R-squared:**	0.421
**Model:**	OLS	**Adj. R-squared:**	0.421
**Method:**	Least Squares	**F-statistic:**	1.618e+06
**Date:**	Tue, 15 Nov 2022	**Prob (F-statistic):**	0.00
**Time:**	22:54:59	**Log-Likelihood:**	-1.0919e+08
**No. Observations:**	12241200	**AIC:**	2.184e+08
**Df Residuals:**	12241196	**BIC:**	2.184e+08
**Df Model:**	3		

	**coef**	**std err**	**z**	**P> |z|**	**[0.025**	**0.975]**
**const**	5041.6989	2.279	2212.697	0.000	5037.233	5046.165
**mu**	-42.7154	0.019	-2195.905	0.000	-42.754	-42.677
**sigma**	-13.8974	0.018	-787.681	0.000	-13.932	-13.863
**excess_states**	-131.1854	0.080	-1643.201	0.000	-131.342	-131.029

**Omnibus:**	2584744.069	**Durbin-Watson:**	0.280
**Prob(Omnibus):**	0.000	**Jarque-Bera (JB):**	5154832.357
**Skew:**	1.274	**Prob(JB):**	0.00
**Kurtosis:**	4.900	**Cond. No.**	214.

Table 26. Final Counts Mean

**Dep. Variable:**	final_counts_sums_median	**R-squared:**	0.395
**Model:**	OLS	**Adj. R-squared:**	0.395
**Method:**	Least Squares	**F-statistic:**	1.424e+06
**Date:**	Tue, 15 Nov 2022	**Prob (F-statistic):**	0.00
**Time:**	22:55:00	**Log-Likelihood:**	-1.0978e+08
**No. Observations:**	12241200	**AIC:**	2.196e+08
**Df Residuals:**	12241196	**BIC:**	2.196e+08
**Df Model:**	3		

	**coef**	**std err**	**z**	**P>|z|**	**[0.025**	**0.975]**
**const**	4855.2822	2.384	2036.872	0.000	4850.610	4859.954
**mu**	-39.7030	0.020	-1938.050	0.000	-39.743	-39.663
**sigma**	-12.8536	0.018	-710.698	0.000	-12.889	-12.818
**excess_states**	-149.4903	0.084	-1786.790	0.000	-149.654	-149.326

**Omnibus:**	2442547.775	**Durbin-Watson:**	0.299
**Prob(Omnibus):**	0.000	**Jarque-Bera (JB):**	4488472.873
**Skew:**	1.259	**Prob(JB):**	0.00
**Kurtosis:**	4.569	**Cond. No.**	214.

Table 27. Final Counts Median

**Dep. Variable:**	final_counts_sums_std	**R-squared:**	0.216
**Model:**	OLS	**Adj. R-squared:**	0.216
**Method:**	Least Squares	**F-statistic:**	8.800e+05
**Date:**	Tue, 15 Nov 2022	**Prob (F-statistic):**	0.00
**Time:**	22:55:01	**Log-Likelihood:**	-9.5805e+07
**No. Observations:**	12241200	**AIC:**	1.916e+08
**Df Residuals:**	12241196	**BIC:**	1.916e+08
**Df Model:**	3		

	**coef**	**std err**	**z**	**P>|z|**	**[0.025**	**0.975]**
**const**	872.4867	0.611	1428.903	0.000	871.290	873.683
**mu**	-9.8917	0.006	-1574.523	0.000	-9.904	-9.879
**sigma**	-2.2708	0.005	-426.064	0.000	-2.281	-2.260
**excess_states**	18.9546	0.027	713.127	0.000	18.903	19.007

**Omnibus:**	4992192.695	**Durbin-Watson:**	0.595
**Prob(Omnibus):**	0.000	**Jarque-Bera (JB):**	21062301.157
**Skew:**	2.024	**Prob(JB):**	0.00
**Kurtosis:**	7.991	**Cond. No.**	214.

Table 28. Final Counts Standard Deviation

2.8 Max Counts

**Dep. Variable:**	max_count_gap	**R-squared:**	0.257
**Model:**	OLS	**Adj. R-squared:**	0.257
**Method:**	Least Squares	**F-statistic:**	1.097e+06
**Date:**	Tue, 15 Nov 2022	**Prob (F-statistic):**	0.00
**Time:**	22:55:31	**Log-Likelihood:**	-5.4500e+07
**No. Observations:**	12241200	**AIC:**	1.090e+08
**Df Residuals:**	12241196	**BIC:**	1.090e+08
**Df Model:**	3		

	**coef**	**std err**	**z**	**P> |z|**	**[0.025**	**0.975]**
**const**	29.7905	0.021	1441.659	0.000	29.750	29.831
**mu**	-0.3500	0.000	-1676.124	0.000	-0.350	-0.350
**sigma**	-0.0806	0.000	-422.635	0.000	-0.081	-0.080
**excess_states**	1.0086	0.001	1017.803	0.000	1.007	1.011

**Omnibus:**	3360665.081	**Durbin-Watson:**	0.618
**Prob(Omnibus):**	0.000	**Jarque-Bera (JB):**	7897406.573
**Skew:**	1.557	**Prob(JB):**	0.00
**Kurtosis:**	5.406	**Cond. No.**	214.

Table 29. Max Counts Range

**Dep. Variable:**	max_count_mad	**R-squared:**	0.209
**Model:**	OLS	**Adj. R-squared:**	0.209
**Method:**	Least Squares	**F-statistic:**	8.843e+05
**Date:**	Tue, 15 Nov 2022	**Prob (F-statistic):**	0.00
**Time:**	22:55:29	**Log-Likelihood:**	-4.2056e+07
**No. Observations:**	12241200	**AIC:**	8.411e+07
**Df Residuals:**	12241196	**BIC:**	8.411e+07
**Df Model:**	3		

	**coef**	**std err**	**z**	**P> |z|**	**[0.025**	**0.975]**
**const**	10.1892	0.008	1313.294	0.000	10.174	10.204
**mu**	-0.1151	7.6e-05	-1513.736	0.000	-0.115	-0.115
**sigma**	-0.0323	6.77e-05	-476.877	0.000	-0.032	-0.032
**excess_states**	0.2646	0.000	841.941	0.000	0.264	0.265

**Omnibus:**	5339043.633	**Durbin-Watson:**	0.618
**Prob(Omnibus):**	0.000	**Jarque-Bera (JB):**	24769883.529
**Skew:**	2.145	**Prob(JB):**	0.00
**Kurtosis:**	8.491	**Cond. No.**	214.

Table 30. Mac Counts Mean Absolute Deviation

**Dep. Variable:**	max_count_max	**R-squared:**	0.486
**Model:**	OLS	**Adj. R-squared:**	0.486
**Method:**	Least Squares	**F-statistic:**	3.797e+06
**Date:**	Tue, 15 Nov 2022	**Prob (F-statistic):**	0.00
**Time:**	22:55:30	**Log-Likelihood:**	-5.6127e+07
**No. Observations:**	12241200	**AIC:**	1.123e+08
**Df Residuals:**	12241196	**BIC:**	1.123e+08
**Df Model:**	3		

	**coef**	**std err**	**z**	**P> |z|**	**[0.025**	**0.975]**
**const**	79.2737	0.024	3333.025	0.000	79.227	79.320
**mu**	-0.7555	0.000	-3329.599	0.000	-0.756	-0.755
**sigma**	-0.2077	0.000	-844.493	0.000	-0.208	-0.207
**excess_states**	-0.5117	0.001	-468.366	0.000	-0.514	-0.510

**Omnibus:**	392617.975	**Durbin-Watson:**	0.447
**Prob(Omnibus):**	0.000	**Jarque-Bera (JB):**	278455.558
**Skew:**	0.265	**Prob(JB):**	0.00
**Kurtosis:**	2.486	**Cond. No.**	214.

Table 31. Max Counts Max

**Dep. Variable:**	max_count_mean	**R-squared:**	0.459
**Model:**	OLS	**Adj. R-squared:**	0.459
**Method:**	Least Squares	**F-statistic:**	2.305e+06
**Date:**	Tue, 15 Nov 2022	**Prob (F-statistic):**	0.00
**Time:**	22:55:26	**Log-Likelihood:**	-5.3925e+07
**No. Observations:**	12241200	**AIC:**	1.078e+08
**Df Residuals:**	12241196	**BIC:**	1.078e+08
**Df Model:**	3		

	**coef**	**std err**	**z**	**P> |z|**	**[0.025**	**0.975]**
**const**	61.6723	0.024	2595.564	0.000	61.626	61.719
**mu**	-0.5337	0.000	-2622.908	0.000	-0.534	-0.533
**sigma**	-0.1509	0.000	-759.686	0.000	-0.151	-0.151
**excess_states**	-1.3529	0.001	-1563.850	0.000	-1.355	-1.351

**Omnibus:**	1379311.651	**Durbin-Watson:**	0.303
**Prob(Omnibus):**	0.000	**Jarque-Bera (JB):**	1952480.074
**Skew:**	0.881	**Prob(JB):**	0.00
**Kurtosis:**	3.852	**Cond. No.**	214.

Table 32. Max Counts Mean

**Dep. Variable:**	max_count_median	**R-squared:**	0.420
**Model:**	OLS	**Adj. R-squared:**	0.420
**Method:**	Least Squares	**F-statistic:**	1.799e+06
**Date:**	Tue, 15 Nov 2022	**Prob (F-statistic):**	0.00
**Time:**	22:55:27	**Log-Likelihood:**	-5.4713e+07
**No. Observations:**	12241200	**AIC:**	1.094e+08
**Df Residuals:**	12241196	**BIC:**	1.094e+08
**Df Model:**	3		

	**coef**	**std err**	**z**	**P> |z|**	**[0.025**	**0.975]**
**const**	59.2008	0.025	2326.351	0.000	59.151	59.251
**mu**	-0.4943	0.000	-2266.073	0.000	-0.495	-0.494
**sigma**	-0.1352	0.000	-655.706	0.000	-0.136	-0.135
**excess_states**	-1.5992	0.001	-1743.071	0.000	-1.601	-1.597

**Omnibus:**	1534130.540	**Durbin-Watson:**	0.352
**Prob(Omnibus):**	0.000	**Jarque-Bera (JB):**	2189897.956
**Skew:**	0.975	**Prob(JB):**	0.00
**Kurtosis:**	3.702	**Cond. No.**	214.

Table 33. Max Counts Median

**Dep. Variable:**	max_count_std	**R-squared:**	0.225
**Model:**	OLS	**Adj. R-squared:**	0.225
**Method:**	Least Squares	**F-statistic:**	9.500e+05
**Date:**	Tue, 15 Nov 2022	**Prob (F-statistic):**	0.00
**Time:**	22:55:28	**Log-Likelihood:**	-4.3390e+07
**No. Observations:**	12241200	**AIC:**	8.678e+07
**Df Residuals:**	12241196	**BIC:**	8.678e+07
**Df Model:**	3		

	**coef**	**std err**	**z**	**P> |z|**	**[0.025**	**0.975]**
**const**	11.6397	0.009	1366.918	0.000	11.623	11.656
**mu**	-0.1330	8.43e-05	-1578.436	0.000	-0.133	-0.133
**sigma**	-0.0361	7.6e-05	-475.483	0.000	-0.036	-0.036
**excess_states**	0.3306	0.000	888.901	0.000	0.330	0.331

**Omnibus:**	4448539.699	**Durbin-Watson:**	0.618
**Prob(Omnibus):**	0.000	**Jarque-Bera (JB):**	15018641.339
**Skew:**	1.876	**Prob(JB):**	0.00
**Kurtosis:**	6.920	**Cond. No.**	214.

Table 34. Max Counts Standard Deviation

2.9 Time of Max

**Dep. Variable:**	max_time_gap	**R-squared:**	0.306
**Model:**	OLS	**Adj. R-squared:**	0.306
**Method:**	Least Squares	**F-statistic:**	2.074e+06
**Date:**	Tue, 15 Nov 2022	**Prob (F-statistic):**	0.00
**Time:**	22:55:51	**Log-Likelihood:**	-5.7646e+07
**No. Observations:**	12241200	**AIC:**	1.153e+08
**Df Residuals:**	12241196	**BIC:**	1.153e+08
**Df Model:**	3		

	**coef**	**std err**	**z**	**P> |z|**	**[0.025**	**0.975]**
**const**	32.7010	0.023	1404.879	0.000	32.655	32.747
**mu**	-0.0734	0.000	-266.694	0.000	-0.074	-0.073
**sigma**	0.3973	0.000	1405.507	0.000	0.397	0.398
**excess_states**	2.1422	0.001	1755.516	0.000	2.140	2.145

**Omnibus:**	291061.139	**Durbin-Watson:**	0.739
**Prob(Omnibus):**	0.000	**Jarque-Bera (JB):**	296568.207
**Skew:**	-0.360	**Prob(JB):**	0.00
**Kurtosis:**	2.750	**Cond. No.**	214.

Table 35. Time of Max Range

**Dep. Variable:**	max_time_mad	**R-squared:**	0.249
**Model:**	OLS	**Adj. R-squared:**	0.249
**Method:**	Least Squares	**F-statistic:**	1.530e+06
**Date:**	Tue, 15 Nov 2022	**Prob (F-statistic):**	0.00
**Time:**	22:55:49	**Log-Likelihood:**	-4.3246e+07
**No. Observations:**	12241200	**AIC:**	8.649e+07
**Df Residuals:**	12241196	**BIC:**	8.649e+07
**Df Model:**	3		

	**coef**	**std err**	**z**	**P> |z|**	**[0.025**	**0.975]**
**const**	9.9347	0.007	1347.274	0.000	9.920	9.949
**mu**	-0.0281	8.46e-05	-332.457	0.000	-0.028	-0.028
**sigma**	0.1133	8.52e-05	1330.470	0.000	0.113	0.114
**excess_states**	0.5338	0.000	1448.866	0.000	0.533	0.535

**Omnibus:**	12903.871	**Durbin-Watson:**	0.851
**Prob(Omnibus):**	0.000	**Jarque-Bera (JB):**	11610.862
**Skew:**	0.037	**Prob(JB):**	0.00
**Kurtosis:**	2.869	**Cond. No.**	214.

Table 36. Time of Max Mean Absolute Deviation

**Dep. Variable:**	max_time_max	**R-squared:**	0.299
**Model:**	OLS	**Adj. R-squared:**	0.299
**Method:**	Least Squares	**F-statistic:**	1.520e+06
**Date:**	Tue, 15 Nov 2022	**Prob (F-statistic):**	0.00
**Time:**	22:55:50	**Log-Likelihood:**	-5.8360e+07
**No. Observations:**	12241200	**AIC:**	1.167e+08
**Df Residuals:**	12241196	**BIC:**	1.167e+08
**Df Model:**	3		

	**coef**	**std err**	**z**	**P> |z|**	**[0.025**	**0.975]**
**const**	45.3687	0.027	1689.473	0.000	45.316	45.421
**mu**	-0.0888	0.000	-301.908	0.000	-0.089	-0.088
**sigma**	0.5825	0.000	1938.772	0.000	0.582	0.583
**excess_states**	1.1336	0.001	879.041	0.000	1.131	1.136

**Omnibus:**	879616.982	**Durbin-Watson:**	0.528
**Prob(Omnibus):**	0.000	**Jarque-Bera (JB):**	1056345.445
**Skew:**	-0.709	**Prob(JB):**	0.00
**Kurtosis:**	2.750	**Cond. No.**	214.

Table 37. Time of Max Max

**Dep. Variable:**	max_time_mean	**R-squared:**	0.322
**Model:**	OLS	**Adj. R-squared:**	0.322
**Method:**	Least Squares	**F-statistic:**	1.822e+06
**Date:**	Tue, 15 Nov 2022	**Prob (F-statistic):**	0.00
**Time:**	22:55:46	**Log-Likelihood:**	-5.4015e+07
**No. Observations:**	12241200	**AIC:**	1.080e+08
**Df Residuals:**	12241196	**BIC:**	1.080e+08
**Df Model:**	3		

	**coef**	**std err**	**z**	**P> |z|**	**[0.025**	**0.975]**
**const**	24.1577	0.019	1294.181	0.000	24.121	24.194
**mu**	-0.0281	0.000	-148.673	0.000	-0.028	-0.028
**sigma**	0.4686	0.000	2308.659	0.000	0.468	0.469
**excess_states**	-0.1939	0.001	-223.001	0.000	-0.196	-0.192

**Omnibus:**	86459.182	**Durbin-Watson:**	0.662
**Prob(Omnibus):**	0.000	**Jarque-Bera (JB):**	85801.509
**Skew:**	0.191	**Prob(JB):**	0.00
**Kurtosis:**	2.850	**Cond. No.**	214.

Table 38. Time of Max Mean

**Dep. Variable:**	max_time_median	**R-squared:**	0.306
**Model:**	OLS	**Adj. R-squared:**	0.306
**Method:**	Least Squares	**F-statistic:**	1.769e+06
**Date:**	Tue, 15 Nov 2022	**Prob (F-statistic):**	0.00
**Time:**	22:55:47	**Log-Likelihood:**	-5.5710e+07
**No. Observations:**	12241200	**AIC:**	1.114e+08
**Df Residuals:**	12241196	**BIC:**	1.114e+08
**Df Model:**	3		

	**coef**	**std err**	**z**	**P> |z|**	**[0.025**	**0.975]**
**const**	21.1694	0.021	1017.129	0.000	21.129	21.210
**mu**	-0.0075	0.000	-35.067	0.000	-0.008	-0.007
**sigma**	0.5155	0.000	2256.946	0.000	0.515	0.516
**excess_states**	-0.3647	0.001	-367.447	0.000	-0.367	-0.363

**Omnibus:**	282341.336	**Durbin-Watson:**	0.850
**Prob(Omnibus):**	0.000	**Jarque-Bera (JB):**	276206.187
**Skew:**	0.336	**Prob(JB):**	0.00
**Kurtosis:**	2.701	**Cond. No.**	214.

Table 39. Time of Max Median

**Dep. Variable:**	max_time_std	**R-squared:**	0.250
**Model:**	OLS	**Adj. R-squared:**	0.250
**Method:**	Least Squares	**F-statistic:**	1.507e+06
**Date:**	Tue, 15 Nov 2022	**Prob (F-statistic):**	0.00
**Time:**	22:55:48	**Log-Likelihood:**	-4.4634e+07
**No. Observations:**	12241200	**AIC:**	8.927e+07
**Df Residuals:**	12241196	**BIC:**	8.927e+07
**Df Model:**	3		

	**coef**	**std err**	**z**	**P> |z|**	**[0.025**	**0.975]**
**const**	11.8444	0.008	1422.078	0.000	11.828	11.861
**mu**	-0.0298	9.53e-05	-312.822	0.000	-0.030	-0.030
**sigma**	0.1264	9.66e-05	1308.440	0.000	0.126	0.127
**excess_states**	0.6074	0.000	1471.395	0.000	0.607	0.608

**Omnibus:**	79714.478	**Durbin-Watson:**	0.801
**Prob(Omnibus):**	0.000	**Jarque-Bera (JB):**	72568.778
**Skew:**	-0.150	**Prob(JB):**	0.00
**Kurtosis:**	2.770	**Cond. No.**	214.

Table 40. Time of Max Standard Deviation

2.10 Min after Max

**Dep. Variable:**	min_post_max_gap	**R-squared:**	0.212
**Model:**	OLS	**Adj. R-squared:**	0.212
**Method:**	Least Squares	**F-statistic:**	1.030e+06
**Date:**	Tue, 15 Nov 2022	**Prob (F-statistic):**	0.00
**Time:**	23:28:45	**Log-Likelihood:**	-5.3282e+07
**No. Observations:**	12241200	**AIC:**	1.066e+08
**Df Residuals:**	12241196	**BIC:**	1.066e+08
**Df Model:**	3		

	**coef**	**std err**	**z**	**P>|z|**	**[0.025**	**0.975]**
**const**	25.3453	0.019	1361.242	0.000	25.309	25.382
**mu**	-0.2934	0.000	-1618.255	0.000	-0.294	-0.293
**sigma**	-0.0379	0.000	-224.221	0.000	-0.038	-0.038
**excess_states**	0.7280	0.001	833.339	0.000	0.726	0.730

**Omnibus:**	4720016.435	**Durbin-Watson:**	0.734
**Prob(Omnibus):**	0.000	**Jarque-Bera (JB):**	17564263.257
**Skew:**	1.956	**Prob(JB):**	0.00
**Kurtosis:**	7.374	**Cond. No.**	214.

Table 41. Min after Max Range

**Dep. Variable:**	min_post_max_mad	**R-squared:**	0.176
**Model:**	OLS	**Adj. R-squared:**	0.176
**Method:**	Least Squares	**F-statistic:**	8.634e+05
**Date:**	Tue, 15 Nov 2022	**Prob (F-statistic):**	0.00
**Time:**	23:28:43	**Log-Likelihood:**	-4.0974e+07
**No. Observations:**	12241200	**AIC:**	8.195e+07
**Df Residuals:**	12241196	**BIC:**	8.195e+07
**Df Model:**	3		

	**coef**	**std err**	**z**	**P>|z|**	**[0.025**	**0.975]**
**const**	8.9245	0.007	1260.068	0.000	8.911	8.938
**mu**	-0.0995	6.71e-05	-1481.687	0.000	-0.100	-0.099
**sigma**	-0.0193	6.08e-05	-316.817	0.000	-0.019	-0.019
**excess_states**	0.1878	0.000	685.193	0.000	0.187	0.188

**Omnibus:**	6359541.303	**Durbin-Watson:**	0.705
**Prob(Omnibus):**	0.000	**Jarque-Bera (JB):**	43173040.063
**Skew:**	2.469	**Prob(JB):**	0.00
**Kurtosis:**	10.762	**Cond. No.**	214.

Table 42. Min after Max Mean Absolute Deviation

**Dep. Variable:**	min_post_max_max	**R-squared:**	0.443
**Model:**	OLS	**Adj. R-squared:**	0.443
**Method:**	Least Squares	**F-statistic:**	2.934e+06
**Date:**	Tue, 15 Nov 2022	**Prob (F-statistic):**	0.00
**Time:**	23:28:44	**Log-Likelihood:**	-5.5406e+07
**No. Observations:**	12241200	**AIC:**	1.108e+08
**Df Residuals:**	12241196	**BIC:**	1.108e+08
**Df Model:**	3		

	**coef**	**std err**	**z**	**P> |z|**	**[0.025**	**0.975]**
**const**	69.1429	0.024	2911.195	0.000	69.096	69.189
**mu**	-0.6393	0.000	-2937.422	0.000	-0.640	-0.639
**sigma**	-0.1623	0.000	-719.202	0.000	-0.163	-0.162
**excess_states**	-0.8403	0.001	-819.848	0.000	-0.842	-0.838

**Omnibus:**	699199.624	**Durbin-Watson:**	0.496
**Prob(Omnibus):**	0.000	**Jarque-Bera (JB):**	827082.529
**Skew:**	0.636	**Prob(JB):**	0.00
**Kurtosis:**	3.053	**Cond. No.**	214.

Table 43. Min after Max Max

**Dep. Variable:**	min_post_max_mean	**R-squared:**	0.424
**Model:**	OLS	**Adj. R-squared:**	0.424
**Method:**	Least Squares	**F-statistic:**	1.662e+06
**Date:**	Tue, 15 Nov 2022	**Prob (F-statistic):**	0.00
**Time:**	23:28:41	**Log-Likelihood:**	-5.3783e+07
**No. Observations:**	12241200	**AIC:**	1.076e+08
**Df Residuals:**	12241196	**BIC:**	1.076e+08
**Df Model:**	3		

	**coef**	**std err**	**z**	**P> |z|**	**[0.025**	**0.975]**
**const**	54.3409	0.024	2249.605	0.000	54.294	54.388
**mu**	-0.4490	0.000	-2179.912	0.000	-0.449	-0.449
**sigma**	-0.1367	0.000	-721.709	0.000	-0.137	-0.136
**excess_states**	-1.5651	0.001	-1804.626	0.000	-1.567	-1.563

**Omnibus:**	2627714.190	**Durbin-Watson:**	0.288
**Prob(Omnibus):**	0.000	**Jarque-Bera (JB):**	5218159.385
**Skew:**	1.299	**Prob(JB):**	0.00
**Kurtosis:**	4.866	**Cond. No.**	214.

Table 44. Min after Max Mean

**Dep. Variable:**	min_post_max_median	**R-squared:**	0.397
**Model:**	OLS	**Adj. R-squared:**	0.397
**Method:**	Least Squares	**F-statistic:**	1.552e+06
**Date:**	Tue, 15 Nov 2022	**Prob (F-statistic):**	0.00
**Time:**	23:28:41	**Log-Likelihood:**	-5.4562e+07
**No. Observations:**	12241200	**AIC:**	1.091e+08
**Df Residuals:**	12241196	**BIC:**	1.091e+08
**Df Model:**	3		

	**coef**	**std err**	**z**	**P> |z|**	**[0.025**	**0.975]**
**const**	52.3302	0.026	2046.006	0.000	52.280	52.380
**mu**	-0.4134	0.000	-1888.762	0.000	-0.414	-0.413
**sigma**	-0.1274	0.000	-646.244	0.000	-0.128	-0.127
**excess_states**	-1.8090	0.001	-1967.189	0.000	-1.811	-1.807

**Omnibus:**	2457182.237	**Durbin-Watson:**	0.332
**Prob(Omnibus):**	0.000	**Jarque-Bera (JB):**	4459586.633
**Skew:**	1.278	**Prob(JB):**	0.00
**Kurtosis:**	4.488	**Cond. No.**	214.

Table 45. Min after Max Median

**Dep. Variable:**	min_post_max_std	**R-squared:**	0.190
**Model:**	OLS	**Adj. R-squared:**	0.190
**Method:**	Least Squares	**F-statistic:**	9.120e+05
**Date:**	Tue, 15 Nov 2022	**Prob (F-statistic):**	0.00
**Time:**	23:28:42	**Log-Likelihood:**	-4.2373e+07
**No. Observations:**	12241200	**AIC:**	8.475e+07
**Df Residuals:**	12241196	**BIC:**	8.475e+07
**Df Model:**	3		

	**coef**	**std err**	**z**	**P> |z|**	**[0.025**	**0.975]**
**const**	10.1663	0.008	1302.176	0.000	10.151	10.182
**mu**	-0.1150	7.49e-05	-1535.672	0.000	-0.115	-0.115
**sigma**	-0.0209	6.86e-05	-304.041	0.000	-0.021	-0.021
**excess_states**	0.2450	0.000	741.117	0.000	0.244	0.246

**Omnibus:**	5529413.732	**Durbin-Watson:**	0.711
**Prob(Omnibus):**	0.000	**Jarque-Bera (JB):**	27457382.233
**Skew:**	2.205	**Prob(JB):**	0.00
**Kurtosis:**	8.863	**Cond. No.**	214.

Table 46. Min after Max Standard Deviation

2.11 Variance in Counts

**Dep. Variable:**	var_count_gap	**R-squared:**	0.157
**Model:**	OLS	**Adj. R-squared:**	0.157
**Method:**	Least Squares	**F-statistic:**	6.825e+05
**Date:**	Tue, 15 Nov 2022	**Prob (F-statistic):**	0.00
**Time:**	23:28:22	**Log-Likelihood:**	-8.7689e+07
**No. Observations:**	12241200	**AIC:**	1.754e+08
**Df Residuals:**	12241196	**BIC:**	1.754e+08
**Df Model:**	3		

	**coef**	**std err**	**z**	**P>|z|**	**[0.025**	**0.975]**
**const**	427.6061	0.340	1256.254	0.000	426.939	428.273
**mu**	-4.1467	0.003	-1422.694	0.000	-4.152	-4.141
**sigma**	-1.9787	0.003	-652.257	0.000	-1.985	-1.973
**excess_states**	2.4104	0.015	163.607	0.000	2.382	2.439

**Omnibus:**	7959364.899	**Durbin-Watson:**	0.925
**Prob(Omnibus):**	0.000	**Jarque-Bera (JB):**	87965486.534
**Skew:**	3.085	**Prob(JB):**	0.00
**Kurtosis:**	14.593	**Cond. No.**	214.

Table 47. Variance in Counts Range

**Dep. Variable:**	var_count_mad	**R-squared:**	0.141
**Model:**	OLS	**Adj. R-squared:**	0.141
**Method:**	Least Squares	**F-statistic:**	6.240e+05
**Date:**	Tue, 15 Nov 2022	**Prob (F-statistic):**	0.00
**Time:**	23:28:21	**Log-Likelihood:**	-7.2936e+07
**No. Observations:**	12241200	**AIC:**	1.459e+08
**Df Residuals:**	12241196	**BIC:**	1.459e+08
**Df Model:**	3		

	**coef**	**std err**	**z**	**P>|z|**	**[0.025**	**0.975]**
**const**	123.5055	0.106	1163.264	0.000	123.297	123.714
**mu**	-1.1673	0.001	-1356.196	0.000	-1.169	-1.166
**sigma**	-0.5759	0.001	-621.061	0.000	-0.578	-0.574
**excess_states**	0.2874	0.004	76.153	0.000	0.280	0.295

**Omnibus:**	9912977.253	**Durbin-Watson:**	0.927
**Prob(Omnibus):**	0.000	**Jarque-Bera (JB):**	237797547.490
**Skew:**	3.861	**Prob(JB):**	0.00
**Kurtosis:**	23.164	**Cond. No.**	214.

Table 48. Variance in Counts Mean Absolute Deviation

**Dep. Variable:**	var_count_max	**R-squared:**	0.210
**Model:**	OLS	**Adj. R-squared:**	0.210
**Method:**	Least Squares	**F-statistic:**	9.719e+05
**Date:**	Tue, 15 Nov 2022	**Prob (F-statistic):**	0.00
**Time:**	23:28:22	**Log-Likelihood:**	-8.8933e+07
**No. Observations:**	12241200	**AIC:**	1.779e+08
**Df Residuals:**	12241196	**BIC:**	1.779e+08
**Df Model:**	3		

	**coef**	**std err**	**z**	**P> |z|**	**[0.025**	**0.975]**
**const**	598.9115	0.390	1535.651	0.000	598.147	599.676
**mu**	-5.4896	0.003	-1698.585	0.000	-5.496	-5.483
**sigma**	-2.6227	0.003	-772.374	0.000	-2.629	-2.616
**excess_states**	-3.1413	0.016	-195.280	0.000	-3.173	-3.110

**Omnibus:**	6843287.047	**Durbin-Watson:**	0.820
**Prob(Omnibus):**	0.000	**Jarque-Bera (JB):**	52374962.931
**Skew:**	2.661	**Prob(JB):**	0.00
**Kurtosis:**	11.623	**Cond. No.**	214.

Table 49. Variance in Counts Max

**Dep. Variable:**	var_count_mean	**R-squared:**	0.231
**Model:**	OLS	**Adj. R-squared:**	0.231
**Method:**	Least Squares	**F-statistic:**	8.706e+05
**Date:**	Tue, 15 Nov 2022	**Prob (F-statistic):**	0.00
**Time:**	23:28:18	**Log-Likelihood:**	-8.0917e+07
**No. Observations:**	12241200	**AIC:**	1.618e+08
**Df Residuals:**	12241196	**BIC:**	1.618e+08
**Df Model:**	3		

	**coef**	**std err**	**z**	**P> |z|**	**[0.025**	**0.975]**
**const**	326.2833	0.232	1409.429	0.000	325.830	326.737
**mu**	-2.6736	0.002	-1598.081	0.000	-2.677	-2.670
**sigma**	-1.2955	0.002	-713.277	0.000	-1.299	-1.292
**excess_states**	-7.5407	0.008	-981.145	0.000	-7.556	-7.526

**Omnibus:**	10410092.720	**Durbin-Watson:**	0.670
**Prob(Omnibus):**	0.000	**Jarque-Bera (JB):**	344310974.964
**Skew:**	4.024	**Prob(JB):**	0.00
**Kurtosis:**	27.704	**Cond. No.**	214.

Table 50. Variance in Counts Mean

**Dep. Variable:**	var_count_median	**R-squared:**	0.194
**Model:**	OLS	**Adj. R-squared:**	0.194
**Method:**	Least Squares	**F-statistic:**	5.904e+05
**Date:**	Tue, 15 Nov 2022	**Prob (F-statistic):**	0.00
**Time:**	23:28:19	**Log-Likelihood:**	-8.1529e+07
**No. Observations:**	12241200	**AIC:**	1.631e+08
**Df Residuals:**	12241196	**BIC:**	1.631e+08
**Df Model:**	3		

	**coef**	**std err**	**z**	**P> |z|**	**[0.025**	**0.975]**
**const**	292.2155	0.247	1182.114	0.000	291.731	292.700
**mu**	-2.2567	0.002	-1295.780	0.000	-2.260	-2.253
**sigma**	-1.1030	0.002	-575.958	0.000	-1.107	-1.099
**excess_states**	-9.1234	0.008	-1135.992	0.000	-9.139	-9.108

**Omnibus:**	11689294.569	**Durbin-Watson:**	0.729
**Prob(Omnibus):**	0.000	**Jarque-Bera (JB):**	548957878.740
**Skew:**	4.705	**Prob(JB):**	0.00
**Kurtosis:**	34.428	**Cond. No.**	214.

Table 51. Variance in Counts Median

**Dep. Variable:**	var_count_std	**R-squared:**	0.148
**Model:**	OLS	**Adj. R-squared:**	0.148
**Method:**	Least Squares	**F-statistic:**	6.491e+05
**Date:**	Tue, 15 Nov 2022	**Prob (F-statistic):**	0.00
**Time:**	23:28:20	**Log-Likelihood:**	-7.4922e+07
**No. Observations:**	12241200	**AIC:**	1.498e+08
**Df Residuals:**	12241196	**BIC:**	1.498e+08
**Df Model:**	3		

	**coef**	**std err**	**z**	**P> |z|**	**[0.025**	**0.975]**
**const**	146.9969	0.122	1200.896	0.000	146.757	147.237
**mu**	-1.4066	0.001	-1384.806	0.000	-1.409	-1.405
**sigma**	-0.6902	0.001	-634.541	0.000	-0.692	-0.688
**excess_states**	0.6448	0.005	134.991	0.000	0.635	0.654

**Omnibus:**	8994793.072	**Durbin-Watson:**	0.916
**Prob(Omnibus):**	0.000	**Jarque-Bera (JB):**	149550983.037
**Skew:**	3.481	**Prob(JB):**	0.00
**Kurtosis:**	18.644	**Cond. No.**	214.

Table 52. Variance in Counts Standard Deviation
